# The burden of chronic rhinosinusitis and its effect on quality of life among patients re-attending an otolaryngology clinic in south western Uganda

**DOI:** 10.1186/s12901-018-0058-z

**Published:** 2018-06-26

**Authors:** Victoria Nyaiteera, Doreen Nakku, Esther Nakasagga, Evelyn Llovet, Elijah Kakande, Gladys Nakalema, Richard Byaruhanga, Francis Bajunirwe

**Affiliations:** 1 Department of Ear, Nose and Throat, Mbarara University of Science and Technology, Mbarara, Uganda; 2 Infectious Disease Research Collaboration, Mbarara, Uganda; 3 Department of Psychology, Mbarara University of Science and Technology, Mbarara, Uganda; 4 Department of Ear, Nose and Throat, Makerere University College of Health Sciences, Kampala, Uganda; 5 Department of Community Health, Mbarara University of Science and Technology, Mbarara, Uganda

**Keywords:** Chronic rhinosinusitis, Quality of life, Sinonasal outcome test 22

## Abstract

**Background:**

Worldwide, the burden of chronic rhinosinusitis (CRS) is variable, but not known in Uganda. CRS has significant negative impact on quality of life (QOL) and as such QOL scores should guide adjustments in treatment strategies. However, most of these studies have been done in the west. Our hypothesis was that QOL scores of the majority of CRS patients in low- to- middle income countries are poorer than those among patients without CRS*.* The aim of this study was to determine the burden of CRS among patients re-attending the Otolaryngology clinic and whether CRS is related to poor QOL.

**Methods:**

A cross sectional study was conducted at Mbarara Regional Referral Hospital Otolaryngology clinic. One hundred and twenty-six adult re-attendees were consecutively recruited. Data was collected using a structured questionnaire and the Sinonasal Outcome Test 22 (SNOT 22) questionnaire measured QOL.

**Results:**

The proportion of re-attendees with CRS was 39.0% (95% CI 30–48%). Majority of CRS patients had poor quality of life scores compared to non-CRS (88% versus 20% *p* < 01). The poor quality of life scores on the SNOT 22 were almost solely as a result of the functional, physical and psychological aspects unique to CRS.

**Conclusions:**

CRS is highly prevalent among re-attendees of an Otolaryngology clinic at a hospital in resource limited settings and has a significant negative impact on the QOL of these patients.

## Background

Chronic rhinosinusitis (CRS) is a condition characterized by the occurrence of two or more of the following signs and symptoms; nasal discharge or post-nasal drip, nasal obstruction, nasal congestion, facial pain, pressure or fullness and decreased sense of smell for a duration of 12 or more weeks with objective findings on either computed tomography or nasal endoscopy [1]. Globally, the prevalence of CRS is variable with occurrence above 10% in Europe and the United States but lower at 2% among populations in sub Saharan Africa [2–4]. In Uganda, anecdotal evidence suggests CRS is a common condition but no formal studies have been conducted to measure disease burden. Preliminary review of records at the otolaryngology Out Patients’ Department (OPD) of Mbarara Regional Referral Hospital (MRRH) in western Uganda showed that 37% of the patients with sinonasal complaints had chronic symptoms evidenced by their re-attendances to the clinic.

Generally, symptoms of CRS interfere with work, leisure and sleep, disrupting the patient’s day-to-day life [5]. This may significantly impact the health related quality of life (HRQoL) of these patients [6]. Moreover, the QoL scores of CRS patients are significantly lower in comparison with the quality of life scores in other common chronic diseases such as congestive heart failure, angina, chronic obstructive pulmonary disease and back pain [5]. Quality of life measures provide a reliable standard as a health outcome, most especially for chronic conditions [6] such as CRS and as such ought to be used routinely in clinical practice. It is therefore imperative that the degree to which a patient’s day-to-day life is affected by CRS be measured and considered more than results of paranasal sinus CT scans or nasal endoscopy when planning or adjusting their management [7]. In order to make appropriate adjustments to the management strategies for CRS based on QoL results, it is necessary to know the factors that may contribute to the observed QoL scores. Documented factors associated with restrictions in HRQoL in CRS patients include; symptom type such as nasal obstruction and postnasal drip, nasal polyposis, comorbidities such as gastro-esophageal reflux disease (GERD), socio-demographic factors such as age and gender and behavioral factors such as smoking [8–10].

CRS patients in south western Uganda make repeated visits to the OPD clinics and consume significant health worker time. They are treated with various medications for prolonged time periods. While these medications are considered to provide relief, there are few studies done to assess the impact of the disease their QoL in Africa. It is also important to identify factors that are associated with poor HRQoL among these patients. This information may be used to make adjustments to CRS treatment in order to improve patient management and satisfaction.

Therefore, the aim of this study was to measure the disease burden of CRS among patients re-attending the Otolaryngology clinic at a tertiary health care facility in a resource limited setting, compare the proportion of patients with poor HRQoL among those with and without CRS and also determine the factors associated with poor HRQoL among these patients.

## Methods

### Study design and site

We conducted a cross sectional study at the otolaryngology clinic at Mbarara Regional Referral Hospital (MRRH) in south western Uganda for three months between June and August 2016. The otolaryngology clinic operates three days a week and attends to an average of three hundred patients per month and serves a catchment population of over 5 million people in the region. We consecutively enrolled study participants who met the eligibility criteria until the required sample size was achieved.

### Eligibility criteria

Patients were recruited into the study if they were re-attending the Otolaryngology clinic at Mbarara Hospital with the recurring symptoms for twelve weeks or more despite previous appropriate treatment as per clinic protocols. The protocols are based on the 2013 Canadian guidelines for management of CRS [11].. Only adult patients aged 18 years of age and over were enrolled. Patients were excluded from the study if they were pregnant because pregnancy related hormonal changes modify sino-nasal mucosal physiology and could therefore mimic CRS symptoms. Participants were also excluded if they could not tolerate the rigid nasal endoscopy (RNE) and there was no evidence of polyps on anterior rhinoscopy. We also excluded patients who were unable to give consent due to mental handicaps or declined participation in the study. Minors were generally excluded because majority of them come to the clinic unaccompanied, yet research guidelines require their caretakers to consent and the youth to provide assent.

### Sample size determination

Sample size calculation was done based on sample size estimation formula for Cross-sectional studies [12]. Using a 95% confidence interval level, we made the assumption that prevalence of CRS among re-attending patients was 7.3%. With an error margin of 5%, the estimated sample size was 126 participants. These participants were then subjected to history and examination, including rigid nasal endoscopy to determine who had CRS and who did not have CRS.

### Data collection procedures

Data was collected using a semi-structured questionnaire and the SNOT 22 tool [13]. The semi-structured questionnaire had two sections. The first part collected information on bio-demographics, presenting complaint, history of presenting complaint, treatment history, comorbidities and patient general health behavior. The second part collected data from physical examination of the sino-nasal region. The QoL assessment was done using the SNOT 22 tool. The SNOT 22 has four domains according to a psychometric analysis done on SNOT 22 responses from patients with CRS [14]. The four domains are rhinologic symptoms, ear and facial symptoms, sleep disturbance and psychological symptoms. Participants rated individual items on a six-point scale (0 - no problem, to 5 - most serious problem). Scores were summed up to obtain the individual domain scores and total scores for each participant. A total score of above 7 was considered an indicator of poor quality of life [15]. Participants were confirmed to be re-attendees by reviewing their previous medical records prior to enrollment.

After participants provided informed consent, the questionnaire was administered and physical examination carried out. The examination included anterior rhinoscopy and the three standard passes of rigid nasal endoscopy. If less than 3 passes were made on a participant with no visible polyps on anterior rhinoscopy, the participant was excluded from the quality of life assessment and therefore not enrolled as we could not then rule out any other endonasal abnormalities that would have been visible on endoscopy. The diagnosis of CRS was therefore based on symptoms and examination findings on rigid nasal endoscopy. Computed tomography was not used because it is an expensive investigation that is not readily available at our center, and Rigid nasal endoscopy is an acceptable objective measure for CRS [3].

### Data analysis

Data were collected in coded form in the questionnaires, entered into Microsoft Excel, cleaned and exported to STATA 11.0 software (College Station, Texas) for analysis.

For the baseline characteristics of study participants such as demographics, summary statistics were generated while proportions were generated for categorical variables. The prevalence of CRS among patients re-attending the otolaryngology clinic at MRRH was determined using the formula; Prevalence = (n/N*100), where n was the total number of re-attendees with CRS and N was the total number of re-attendees enrolled.

The individual domain scores and total SNOT 22 score were obtained for each participant. A SNOT 22 score of above 7 was considered poor QoL and a score of 7 and below as considered normal [15]. The proportion of patients with a score of above 7in each category of CRS was calculated. We conducted a chi square test to compare the proportion of patients with good QOL scores in the CRS and non- CRS categories. We also compared the mean SNOT 22 scores of patients with and without CRS, and using an independent samples t-test, determined whether the mean scores differed significantly. The same analysis was conducted to compare the mean scores derived within the domains, to determine whether the individual domain scores of SNOT 22 differed among patients with and without CRS.

To determine the association between poor quality of life and the clinical and non-clinical factors associated with the disease specific quality of life, we conducted a Univariate logistic regression analysis. The outcome variable was the total QOL score on SNOT 22 measured as a dichotomous variable. Variables with *p* values less than 0.05 and those of relevant clinical significance were entered into a multiple logistic regression model to build a predictive model for poor QOL. The crude odds ratios were calculated to eliminate confounders after which all statistically significant factors (*p* < 0.05) and those of biological significance were considered for multivariate analysis. We reported the adjusted odds ratios with 95% confidence intervals.

### Ethical consideration

Ethical approval was given by the Mbarara University of Science and Technology Research and Ethics Committee (MUST-REC) study number 03/05–16. Signed informed consent was obtained from all study participants in English or the common local language (Runyankore) before enrollment into the study. Participants were informed that they were free to withdraw their consent at any point during the course of the study and that this would not affect their care in any way. All participants received appropriate management according to applied guidelines in the MRRH Otolaryngology clinic. Study participants were given unique identifiers to ensure confidentiality. Any information that could lead to identification of a participant was not collected.

## Results

### Prevalence of CRS and socio-demographic and behavioral characteristics of patients

One hundred and twenty six (*n* = 126) adult re-attendees at the MRRH Otolaryngology clinic were enrolled into this study. Overall 49 of the 126 patients or 38.8% had CRS. The median age of the group was 42 years with an inter quartile range (IQR) of 22–59, and 57% of respondents were female. The results of the baseline characteristics are shown in Table 1 below.

**Table 1:** Socio-demographic and behavioral characteristics of patients stratified by CRS status among patients attending ENT clinic at Mbarara, Uganda

VARIABLE	CRS *n* = 49 Frequency (%)	No CRS *n* = 77 Frequency (%)	*p* value
Age categories in years			
18–35	17 (34.69)	30 (38.96)	0.86
36–59	24 (48.98)	34 (44.16)	
60–80	8 (16.33)	13 (16.88)	
Age (continuous)			
median, IQR	43 (30–53)	42 (29–54)	0.87
Gender			
Male	17 (34.69)	36 (46.75)	0.18
Female	32 (65.31)	41 (53.25)	
Formal Education			
None	14 (28.57)	13 (16.88)	0.32
Primary	12 (24.49)	27 (35.06)	
Secondary	19 (18.37)	18 (23.38)	
Tertiary	14 (28.57)	19 (24.68)	
Monthly income			
None	26 (53.06)	25 (32.47)	0.02
>/=USD 15	23 (46.94)	52 (67.53)	
History of ever smoking			
No	38 (77.50)	63 (81.82)	0.56
Yes	11 (22.45)	14 (18.18)	
Did not complete course of treatment			
No	43 (87.76)	72 (93.51)	0.27
Yes	6 (12.24)	5 (6.49)	

*CRS* Chronic rhinosinusitis, *IQR* Inter quartile range, *USD* United States Dollars

With data broken down by CRS status, there was no significant difference in terms of distribution of age, gender, education, smoking history and completion of treatment. The two groups of patients were similar on many baseline demographic characteristics. However, almost 20% of the study participants had a history of ever smoking and of these 44.0% had CRS. Patients with no CRS were more likely to be in the higher income categories compared to those without CRS and this difference was statistically significant.

In general, almost 40% of patients re-attending the ENT clinic at Mbarara Hospital had CRS and the two group patients were comparable regarding the distribution of most socio-demographic features.

### Clinical characteristics of respondents stratified by CRS status

Overall, 46% (*n* = 58) of the respondents had a SNOT22 score of above 7, an indicator of poor quality of life. Among the patients with CRS, 43 (87.8%) had a poor quality of life while among those without CRS only 15 (19.4%) had a poor quality of life. Analysis of the clinical characteristics showed that 25.4% (*n* = 32) of all the respondents had symptoms of allergy and only 6.4% (*n* = 8) had symptoms of reflux for more than 12 weeks. This was taken to be indicative of gastroesophageal reflux disease (GERD). Patients with CRS were also more likely to have nasal polyps, nasal discharge, nasal mucosal edema and nasal crusts. In general, patients with CRS had a poorer quality of life and were more likely to have accompanying intra-nasal abnormalities.

### Proportion of respondents with poor QOL scores on SNOT 22 among patients with CRS and those without CRS

Among patients with CRS, 43 (74.1%) had a poor QoL score on SNOT 22 compared to only 15 (25.9%) among those without CRS. Similarly, among the patients with CRS, only 6 (8.82%) had normal QoL scores while the majority (*n* = 62 or 91.2%) among patients that did not have CRS had normal QoL scores. The statistical test to compare the two groups showed a significant difference between these proportions (chi square test for independence, *p* value< 0.0001).

Overall, about two thirds of patients with CRS reported poor QOL compared to only one third of those without CRS indicating patients with CRS are significantly more likely to report poorer QOL.

### Comparison of mean scores of SNOT 22 by CRS status

**Fig. 1:**
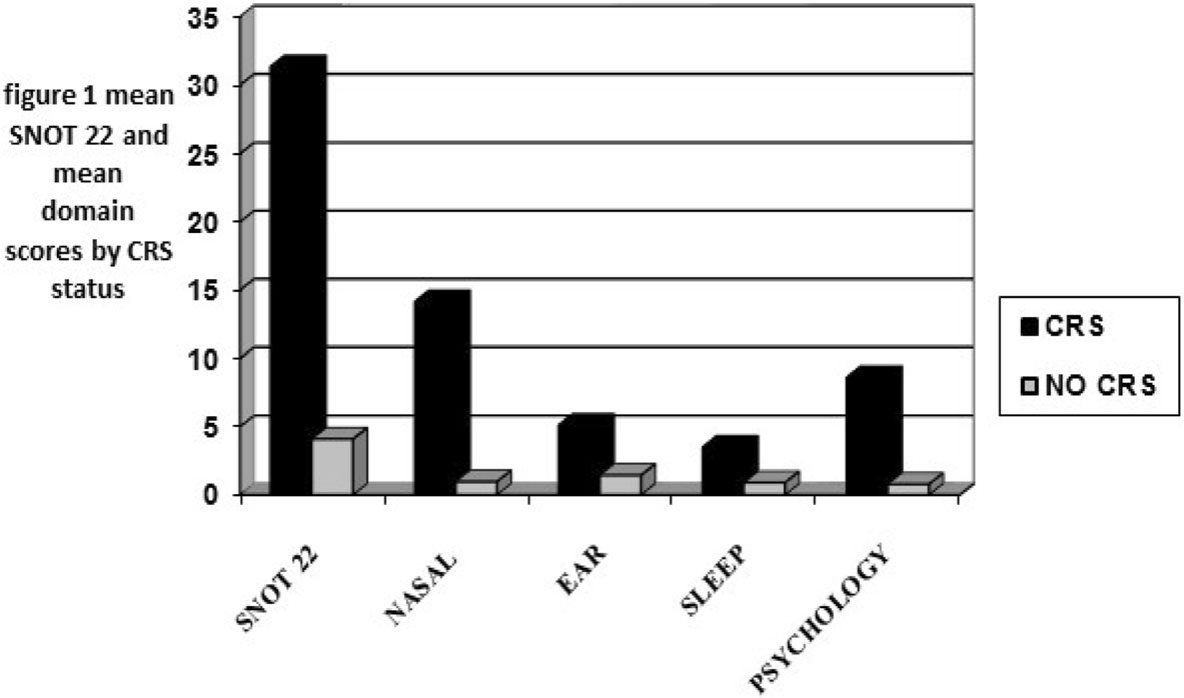
Mean SNOT 22 scores and mean domain scores by CRS status

Overall, both the SNOT 22 and domain mean scores were higher in patients with CRS, signifying poorer quality of life among these patients, compared to those without CRS. These results are shown in Table 2 below. Among the respondents with CRS, the mean SNOT 22 score was 31.4 (95% CI 25.26–37.44) while that of patients without CRS was 4.10 (95% CI 2.35–5.86). The data on mean scores in each domain are shown in Fig. 1 below. The domains with the highest mean scores were nasal symptoms (14.1, 95% CI =11.68–16.61) among CRS patients and ear symptoms 1.44 (95% CI 0.92–1.96), among those without CRS. The domains with the lowest mean scores for patients with CRS and those without CRS were sleep disturbance (3.5, 95% CI = 1.96–5.0) and the psychological domain 0.79 (95% CI 0.10–1.48). The *p* values generated from this comparison of the mean quality of life scores were all significant (*p* < 0.001).

**Table 2:** Clinical characteristics of respondents stratified by CRS status, among patients attending ENT clinic, Mbarara, Uganda

VARIABLE	CRS n = 49 Frequency (%)	No CRS n = 77 Frequency (%)	*p* value
Quality of life on SNOT 22			
Good	6 (12.24)	62 (80.52)	< 0.01
Poor	43 (87.76)	15 (19.48)	
History of nasal trauma or nasal surgery			
No	40 (81.63)	70 (90.91)	0.13
Yes	9 (18.37)	7 (9.07)	
GERD			
No	45 (91.84)	73 (94.18)	0.51
Yes	4 (8.16)	4 (5.19)	
Nasal polyps			
No	37 (75.51)	76 (98.70)	< 0.01
Yes	12 (24.49)	1 (1.30)	
Nasal discharge			
No	14 (28.57)	73 (94.81)	< 0.01
Yes	35 (71.43)	4 (5.19)	
Nasal mucosal edema			
No	17 (34.69)	72 (97.40)	< 0.01
Yes	32 (65.31)	2 (2.60)	
Nasal crusts			
No	42 (85.71)	75 (97.40)	0.01
Yes	7 (14.29)	2 (2.60)	
Number of CRS symptoms:			
0	2 (4.08)	50 (64.94)	< 0.01
1	1 (2.04)	18 (23.38)	
> 1	46 (93.88)	9 (11.69)	

*SNOT 22* Sinonasal Outcome Test 22, *GERD* Gastroesophageal Reflux Disease

Overall, patients with CRS had higher mean SNOT22 scores suggesting significantly poorer QoL compared to those without CRS.

### Factors associated with poor quality of life scores on SNOT 22 questionnaire

Table 3 shows the factors that were associated with a poor QoL in a crude analysis. Gender, age category, in-adherence to medication and presence of GERD symptoms, did not have a significant relationship with QoL. Patients with a monthly income over 50,000 Ugx were less likely to have poor QoL compared to patients that did not have a monthly income. A patient with a primary education was less likely to have a poor QoL compared to another with no formal education. Similarly, a secondary school and tertiary education made one times less likely to have a poor QoL. A history of smoking, whether past or current, was associated with a 2.5-fold increase in the odds of having a poor QoL compared to those that had never smoked before. Having allergies was associated with a 2.9 fold increased odds of having a poor quality of life compared to absence of allergies. Other variables associated with increased likelihood of poor quality of life were endo-nasal including nasal polyps **(**OR = 17.5, *p* = 0.01), nasal discharge (OR = 24.4, *p* < 0.01), and nasal mucosal edema (OR = 15.51, p < 0.01).

**Table 3:** Bivariate analysis for socio-demographic, behavioral and clinical factors associated with a poor quality of life (QoL) among patients attending ENT outpatients, Mbarara, Uganda

VARIABLE	Good QoL (%)	Poor QoL (%)	COR (95% CI)	*p* value
Age category in years				
18–35	28 (41.2)	19 (32.8)	1.0	
36–59	30 (44.1)	28 (48.3)	1.38 (0.6–4.6)	0.42
60–80	10 (14.7)	11 (19.0)	1.62 (0.6–4.6)	0.36
Gender				
female	40 (58.8)	33 (56.9)	1.0	
male	28 (41.2)	25 (43.1)	1.08 (0.5–2.2)	0.83
Monthly income				
< USD 15	20 (29.4)	31 (53.5)	1.0	
>/=USD 15	48 (70.6)	27 (46.6)	0.36 (0.2–0.8)	0.01*
Formal Education				
None	9 (13. 2)	18 (31.0)	1.0	
Primary	23 (33.8)	16 (27.6)	0.35 (0.1–1.0)	0.04*
Secondary	17 (25.0)	10 (17.2)	0.29 (0.1–1.0)	0.03*
Tertiary	19 (27.9)	14 (24.1)	0.37 (0.2–0.8)	0.06
History of ever smoking:				
No	59 (86.8)	42 (72.4)	1.0	
Yes	9 (13.2)	16 (27.6)	2.50 (1.0–6.2)	0.05
CRS				
No	62 (91.2)	15 (25.9)	1.0	
Yes	6 (8.8)	43 (74.1)	29.62(10.6–82.4)	0.01*
Allergy symptoms				
No	57 (83.3)	37 (63.8)	1.0	
Yes	11 (16.2)	21 (36.2)	2.94 (1.3–6.8)	0.01*
GERD				
No	64 (94.1)	54 (93.1)	1.0	
Yes	4 (5.9)	4 (6.9)	1.1 (0.3–5.0)	0.82
Nasal polyps:				
No	67 (98.5)	46 (79.3)	1.0	
Yes	1 9 1.5)	12 (20.7)	17.5(2.2–139.0)	0.01*
Nasal discharge:				
No	64 (94.1)	23 (37. 9)	1.0	
Yes	4 (5.9)	35 (60.3)	24.40 (7.8–76.1)	0.01*
Nasal mucosal edema				
No	63 (92.7)	26 (44.8)	1.0	
Yes	5 (7.4)	32 (55.2)	15.51 (5.5–44.2)	0.01*
Number of CRS symptoms:				
0	46 (67.7)	6 (10.3)	1.0	
1	12 (17.7)	7 (12.1)	4.47 (1.3–15.8)	0.02*
> 1	10 (14.7)	45 (77.6)	34.5(11.6–102.9)	0.01*

*QoL* Quality of life, *COR* Crude odds ratio

*p value =/< 0.05

The main exposure variable, CRS, was significantly associated with poor quality of life, with a 29.6 fold increase in the odds of having a poor quality of life score (OR = 29.6, *p* < 0.00).

Socio-demographic and behavioral factors that were significantly associated with QoL (*p* < 0.05) from bivariate analysis included monthly income, in-adherence to treatment and a history of smoking. Clinical factors that were significantly associated with QoL were CRS status, allergies, nasal polyps, nasal discharge, mucosal edema, osteo-meatal complex abnormalities and number of symptoms. These were included in the multivariate model together with GERD because it is of biological importance as modifier of disease specific quality of life in CRS.

The results of the multivariable regression are presented in Table 4. Here, patients with CRS had an 8.94-fold increase in the odds of having poor quality of life (OR = 8.94, *p* < 0.01), compared to those without CRS. Persons with nasal discharge had a 7.15 fold increase in the odds of having poor QoL compared to those without nasal discharge (OR = 7.15, *p* = 0.01).

**Table 4:** Factors associated with poor quality of life on SNOT 22 after controlling for confounders

VARIABLE	Crude odds ratio (95% CI)	Adjusted odds ratio (95% CI)	*p* value
CRS			
No			
Yes	29.62 (10.6–82.4)	8.94 (2.4–33.8)	0.00^a^
Polyps			
No			
Yes	17.5 (2.2–139.0)	2.14 (0.1–53.3)	0.64
Nasal discharge			
No			
Yes	24.40 (7.8–76.1)	7.15 (1.6–32.8)	0.01^a^
Allergy			
No			
symptoms			
Yes	2.94 (1.3–6.8)	1.94 (0.5–7.9)	0.35
GERD			
No			
Yes	1.1 (0.3–5.0)	0.69 (0.1–5.9)	0.74
History of smoking			
No			
Yes	2.50 (1.0–6.2)	3.65 (0.9–15.1)	0.07
Monthly income			
< USD 15			
>/=USD 15	0.36 (0.2–0.8)	0.48 (0.2–1.5)	0.20

*CI* Confidence interval, ^a^significant at 0.05 level

## Discussion

### Prevalence of CRS among re-attendees at the MRRH ENT clinic

The prevalence of CRS among patients re-attending the ENT clinic in a resource limited setting was 39%. Our study results are generalizable to studies that have enrolled patients re-attending the ENT clinic in resource limited settings. However, very few studies have been done to measure CRS in this population, and not only in resource limited setting but globally. When compared to the prevalence of CRS in the general population surveys, the results are stunningly different. For instance a cross sectional survey of 19 European countries by the GA2LEN network of excellence showed variable prevalence of CRS ranging from 6.9% (95% CI 5.8–8.2%) to 27.1% (95% CI 25.0–29.3%) in Germany [2], the maximum prevalence measured was 27.1%which is still be lower than that in our study. In an African setting, Iseh and Makusidi recruited all new patients with the diagnosis of rhinosinusitis over a 2-year period and found a prevalence of CRS of 7.3% at a teaching hospital in north western Nigeria among patients attending an ENT clinic [16].

Clearly, the high prevalence in our study is because the respondents in our study were re-attendees, a high-risk group of patients with chronic ailments of which CRS is one. Secondly, we recruited patients at a large government health facility that attracts patients from a mostly low socioeconomic status because of free services. This category of patients is known to be at high risk for CRS [17] and our data also confirms this. The ENT clinic at our facility also serves a larger population than most Regional Referral Hospitals in Uganda. We serve patients from 10 districts of South Western Uganda and neighboring countries of Rwanda, Burundi, Southern Tanzania and The Democratic Republic of Congo. This might further explain the high prevalence we found in comparison to the studies reviewed [2–4].

### Proportion of patients with poor HRQoL among those with and without CRS

Generally, CRS patients have poorer QoL compared to healthy individuals [18–20]. Our study showed the same as patients with CRS had poorer Health Related Quality of life, compared to those without CRS. The domain with the highest mean score among CRS patients was the nasal symptom domain. This might be because the aspects of QoL assessed in this domain relate to the mucosal inflammatory and ostial obstructive mechanisms within CRS. We found that the psychological domain had the second highest mean score among the CRS patients and attribute this to possibly unexplored sources of psychological stress such as financial struggles in this low socio-economic setting.. Browne et al., found the highest mean score in CRS patients in the nasal symptom domain, followed closely by the mean psychological score [14], a finding similar to that in our study.

Non-CRS patients scored highest in the ear/ facial symptom domain. We attribute this to the fact that the majority of patients seen in the MRRH otolaryngology clinic have ear related conditions from a review of the OPD records. This likely resulted in the majority of our non-CRS respondents having ear related complaints, thus reporting poorer quality of life scores in the ear symptom domain. Mean scores and trends in the SNOT 22 domains for non- CRS groups is variable across studies possibly because there is wide diversity in the non-CRS groups recruited [14, 21, 22].

Overall, both the total and mean domain scores were higher in the CRS patients compared to respondents without CRS and this is in keeping with results from studies done elsewhere. There is however little similarity in the scores from the non-CRS population between our study and studies done elsewhere.

### Factors associated with poor quality of life scores

From the bivariate analysis, poor quality of life scores were generally significantly associated with the endonasal factors except septal deviation. Having a secondary education and a monthly income of over USD 15 appeared to confer protection from poor quality of life in CRS.

We expected to find a significant association between female gender and poor quality of life but this was not true. Males tend to seek health care when they have worse symptom scores compared to females and males were also more likely to be smokers. Although females tend to report poorer QoL [23], one may argue that male health seeking behavior and smoking may have wiped out the difference. However, this may not be entirely true since we adjusted for these differences. Ference et al., reviewed six studies on gender differences in self-reported quality of life among CRS patients. They concluded that the influence of gender on quality of life seems to be restricted primarily to the general aspects of quality of life, whereas the disease-specific health-related quality of life is not different between genders [23].

The lack of association between GERD symptoms and both CRS and quality of life on the SNOT 22 in our study is not supported by previous findings. Patients with GERD symptoms have been shown to have a reduced nose and sinus-related quality of life [24] and having GERD symptoms increases the mean SNOT-22 score in patients with CRS by 15.7 (95% CI, 6.5–24.9) [25]. We suspect that this discrepancy in findings may be because only 8 respondents in our study had GERD symptoms, with 4 of them having a poor quality of life.

Our study revealed that having a formal education was protective in CRS related quality of life. A study by Kilty et al., found that having a post-secondary education was significantly associated with low self-reported sinus symptom scores in CRS patients [26]. It is possible that for our population, patients with a formal education possibly understand prescription instructions better than those without a formal education and medical personnel find more ease in explaining disease processes to formally educated persons. This means that an educated patient might have realistic health expectations during the course of their treatment and are psychologically better equipped to manage their CRS symptoms.

Patients with a monthly income of over USD 15 were less likely to have poor quality of life. Also CRS occurred less frequently among patients with a monthly income. USD 15 is averagely sufficient to purchase a month’s supply of CRS medication with some left over to cater to other basic needs of a patient in this population. Although we could not find studies that evaluated income level and quality of life in CRS patients, Pilan et al., found that CRS was significantly more prevalent in low-income groups [17]. The ability to purchase prescribed medication that would relieve CRS symptoms and thus result in better quality of sleep and psychological wellbeing may account for this relationship.

Because endonasal abnormalities result in persistent symptoms, they may have contributed greatly to increased nasal symptom and sleep disturbance scores for our patients. Nasal discharge is a major symptom of CRS, therefore it would seem imperative that it be associated with a poor Health Related Quality of Life in our patient sample.

In the multivariate analysis, only CRS and nasal discharge were found to be significantly associated with poor health related quality of life. Persons with CRS were almost nine times more likely to have poor quality of life compared to persons without CRS. The results agree with findings from other studies using SNOT22 among CRS patients [18–20].

Our study has some limitations. First, the SNOT-22 questionnaire has not been validated in any of the indigenous languages of Uganda, however, has been used elsewhere in Africa with similar cultural setting as Uganda. We used professional translation and hence language minimally affected the validity of the results obtained from the Quality of life assessment.

Second, the lack of CT scans limited our ability to accurately diagnose CRS. However we are confident that the symptoms and endoscopic findings were adequate to make a diagnosis of CRS as per the definition by Rosenfeld and Cornelius [3, 27].

Our study has some strength. Most quality of life studies have been done in regions that have significant seasonal variations compared to southwestern Uganda. The strength of our study is that it provides data from a region with less seasonal variation, such as sub Saharan Africa, for which information on quality of life in CRS is scarce.

## Conclusions

In conclusion, the prevalence of chronic rhinosinusitis among our patients re-attending the ENT clinic is relatively high. Majority of patients with CRS in this resource limited settinghave significantly reduced disease specific QOL compared to those without CRS.

The poor quality of life scores on SNOT 22 appear to be almost solely as a result of the functional, physical and psychological aspects of chronic rhinosinusitis that the SNOT 22 evaluates.

Quality of life assessment should therefore be included in the routine evaluation of patients with CRS in low-to-middle income countries, and the quality of life scores used as one of the indices, alongside clinical findings to assess treatment strategies. This will ensure holistic care of these patients. Further studies need to be done to evaluate the impact of treatments available in low resource settings on the quality of life in CRS patients.

## Abbreviations

CRS: Chronic Rhinosinusitis
FREC: Faculty of Medicine Research Ethical Committee
GERD: Gastroesophageal Reflux Disease
HRQoL: Health-related Quality of Life
IRB: Institutional Review Board
MRRH: Mbarara Regional Referral Hospital
MUST: Mbarara University of Science and Technology
QoL: Quality of Life
RNE: Rigid nasal endoscopy
SNOT-22: Sinonasal Outcome Test 22

## References

[1] Fokkens WJ, Lund VJ, Mullol J, Bachert C, Alobid I, Baroody F (2012). EPOS 2012: European position paper on rhinosinusitis and nasal polyps 2012. A summary for otorhinolaryngologists. Rhinology.

[2] Hastan D, Fokkens W, Bachert C, Newson R, Bislimovska J, Bockelbrink A (2011). Chronic rhinosinusitis in Europe–an underestimated disease. A GA2LEN study. Allergy.

[3] Cornelius RS, Martin J, Wippold FJ, Aiken AH, Angtuaco EJ, Berger KL (2013). ACR appropriateness criteria sinonasal disease. J Am Coll Radiol.

[4] Mainasara MG, Labaran AS, Kirfi AM, Fufore MB, Fasunla AJ, Sambo GU. Clinical profile and management of chronic rhinosinusitis among adults in North-Western Nigeria. Magn Resonance Imaging (MRI). 2015; 9:11–2.

[5] Metson RB, Gliklich RE (2000). Clinical outcomes in patients with chronic sinusitis. Laryngoscope.

[6] Fitzpatrick R, Fletcher A, Gore S, Jones D, Spiegelhalter D, Cox D (1992). Quality of life measures in health care. I: applications and issues in assessment. BMJ.

[7] Schalek P. Rhinosinusitis-its impact on quality of life: INTECH open access Publisher; 2011.

[8] Damm M, Quante G, Jungehuelsing M, Stennert E (2002). Impact of functional endoscopic sinus surgery on symptoms and quality of life in chronic rhinosinusitis. Laryngoscope.

[9] Alobid I, Benitez P, Bernal-Sprekelsen M, Roca J, Alonso J, Picado C (2005). Nasal polyposis and its impact on quality of life: comparison between the effects of medical and surgical treatments. Allergy.

[10] Baumann I, Blumenstock G (2005). Impact of gender on general health-related quality of life in patients with chronic sinusitis. Am J Rhinol.

[11] Kaplan A (2013). Canadian guidelines for chronic rhinosinusitis: clinical summary. Can Fam Physician.

[12] Kelsey J, Whittemore A, Evans A, Thompson W (1996). Methods of sampling and estimation of sample size. Methods Observational Epidemiol.

[13] Piccirillo JF, Merritt MG, Richards ML (2002). Psychometric and clinimetric validity of the 20-item Sino-nasal outcome test (SNOT-20). Otolaryngol Head Neck Surg.

[14] Hopkins C, Gillett S, Slack R, Lund V, Browne J (2009). Psychometric validity of the 22-item Sinonasal outcome test. Clin Otolaryngol.

[15] Gillett S, Hopkins C, Slack R, Browne J (2009). A pilot study of the SNOT 22 score in adults with no sinonasal disease. Clin Otolaryngol.

[16] Iseh K, Makusidi M. Rhinosinusitis: a retrospective analysis of clinical pattern and outcome in north western Nigeria. Ann Afr Med. 2010;9(1)22–26 10.4103/1596-3519.62620 20418645

[17] Pilan RR, FdR P, Bezerra TF, Mori RL, Padua FG, Bento RF (2012). Prevalence of chronic rhinosinusitis in Sao Paulo. Rhinology.

[18] Browne JP, Hopkins C, Slack R, Cano SJ (2007). The Sino-nasal outcome test (SNOT): can we make it more clinically meaningful?. Otolaryngol Head Neck Surg.

[19] Lange B, Holst R, Thilsing T, Baelum J, Kjeldsen A (2013). Quality of life and associated factors in persons with chronic rhinosinusitis in the general population: a prospective questionnaire and clinical cross-sectional study. Clin Otolaryngol.

[20] Kosugi EM, Chen VG, da Fonseca VMG, Cursino MMP, Neto JAM, Gregório LC (2011). Translation, cross-cultural adaptation and validation of SinoNasal outcome test (SNOT)-22 to Brazilian Portuguese. Braz J Otorhinolaryngol.

[21] Jalessi M, Farhadi M, Kamrava SK, Amintehran E, Asghari A, Hemami MR, Mobasseri A, Masroorchehr M. The reliability and validity of the persian version of sinonasal outcome test 22 (snot 22) questionnaires. Iran Red Crescent Med J. 2013;15(5):404–8. 10.5812/ircmj.7937 PMC3838650 24349728

[22] Mascarenhas JG, VMGd F, Chen VG, Itamoto CH, CAPd S, Gregório LC, et al. Results in longo prazo da cirurgia endoscópica nasossinusal no tratamento da rinossinusite crônica com e sem polipos nasais. Braz J Otorhinolaryngol. 2013; 79 (3): 306–11.

[23] Ference EH, Tan BK, Hulse KE, Chandra RK, Smith SB, Kern RC (2015). Commentary on gender differences in prevalence, treatment, and quality of life of patients with chronic rhinosinusitis. Allergy & Rhinology.

[24] Katle E-J, Hart H, Kjærgaard T, Kvaløy JT, Steinsvåg SK (2012). Nose-and sinus-related quality of life and GERD. Eur Arch Otorhinolaryngol.

[25] Bohnhorst I, Jawad S, Lange B, Kjeldsen J, Hansen JM, Kjeldsen AD (2015). Prevalence of chronic rhinosinusitis in a population of patients with gastroesophageal reflux disease. Am J Rhinol Allergy.

[26] Kilty SJ, McDonald JT, Johnson S, Al-Mutairi D (2011). Socioeconomic status: a disease modifier of chronic rhinosinusitis?. Rhinology.

[27] Rosenfeld RM, Piccirillo JF, Chandrasekhar SS, Brook I, Kumar KA, Kramper M (2015). Clinical Practice Guideline (Update) Adult Sinusitis. Otolaryngol Head Neck Surg.

